# A New Tetrahydrofuran Lignan Diglycoside from *Viola tianshanica* Maxim

**DOI:** 10.3390/molecules181113636

**Published:** 2013-11-04

**Authors:** Yan Qin, Chengle Yin, Zhihong Cheng

**Affiliations:** 1 Department of Pharmacognosy, School of Pharmacy, Fudan University, Shanghai 201203, China; 2 Key Laboratory of Modern Chinese Preparation, Jiangxi University of Traditional Chinese Medicine, Ministry of Education of China, Nanchang 330004, China

**Keywords:** *Viola tianshanica*, lignan, tianshanoside A, cytotoxicity

## Abstract

A new lignan glycoside, tianshanoside A (**1**), together with a known phenylpropanoid glycoside, syringin (**2**) and two known lignan glycosides, picraquassioside C (**3**), and aketrilignoside B (**4**), were isolated from the whole plant of *Viola tianshanica* Maxim*.* The structure of the new compound was elucidated by extensive NMR (^1^H, ^13^C, COSY, HSQC, HMBC and ROESY) and high resolution mass spectrometry analysis. The three lignans **1**, **3**, and **4** did not exhibit significant cytotoxicity against human gastric cancer Ags cells or HepG2 liver cancer cells. This is the first report of the isolation of a lignan skeleton from the genus *Viola* L.

## 1. Introduction

**Figure 1:**
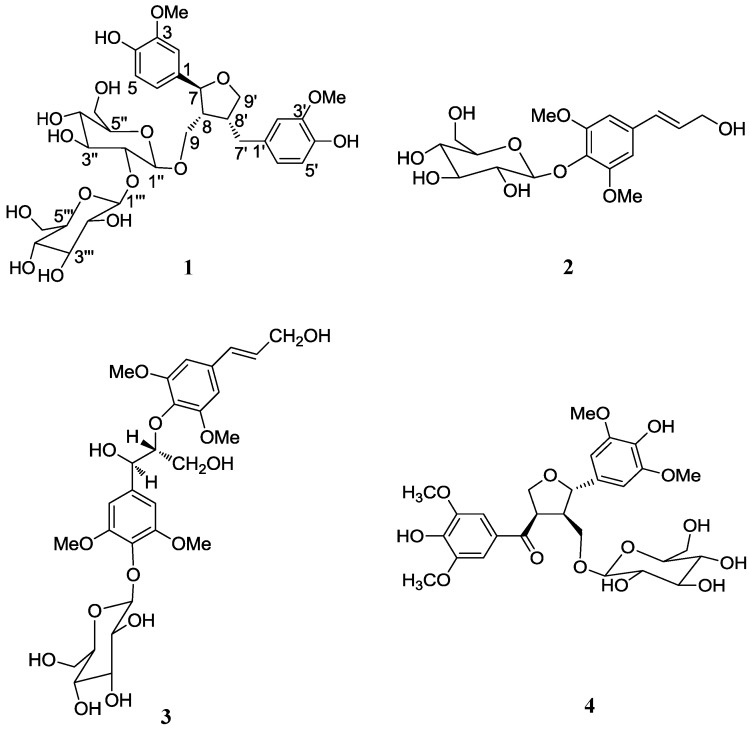
Structures of compounds **1**–**4**.

*Viola tianshanica* Maxim (Violaceae) is a perennial herb widely distributed in Central Asia, especially in the Xinjiang Uygur Autonomous Region (XUAR) of China [1]. The whole herb, including the roots, have been used in traditional Uygur medicines as an antifebrile and detoxicating agent for the treatment of fever, headache, pharyngalgia and acute pyogenic infections such as boils, furuncles and carbuncles [2]. Modern pharmacological studies have demonstrated that the extracts of *V. tianshanica* have anti-inflammatory [3], anti-bacterial [4] and anti-oxidative [5] activities. It is used in XUAR as a substitute for *Viola yedoensis*. Very little is known about the phytochemical composition of *V. tianshanica* with the exception of several flavonol-*O*-glycosides [6] and cyclotides [7] that have been reported. As part of our continued investigation of the constituents of *V. yedoensis* and its substitutes [8,9], a phytochemical study of the ethanolic extract of the herb was thus carried out. Herein, we report the isolation and structural elucidation of a new lignan glycoside, tianshanoside A (**1**), along with three known glycosidic lignan derivatives, syringin (**2**) [10], picraquassioside C (**3**) [11], and aketrilignoside B (**4**) [12] (Figure 1), from *V*. *tianshanica*. Compounds **1**, **3**, and **4** were evaluated for their cytotoxicity against the human gastric cancer (Ags) and liver cancer HepG2 cell lines.

## 2. Results and Discussion

Repeated column chromatography of the ethyl acetate fraction of the ethanolic extract of *V. tianshanica* led to the isolation of a new tetrahydrofuran lignan diglycoside (**1**). Compound **1** was obtained as a white amorphous powder and possessed the molecular formula of C_32_H_44_O_16_ as evidenced by a HRESIMS peak at *m*/*z* 707.2531 [M + Na]^+^ in combination with its ^1^H-NMR, ^13^C-NMR and DEPT spectra. Maximum absorption bands at 230 and 280 nm in the UV spectrum suggested the existence of conjugated groups in **1**. The IR spectrum exhibited absorption bands for free hydroxyl groups (3387 cm^−1^) and benzene rings (1637 and 1513 cm^−1^). Detailed analysis of ^1^H-NMR (Table 1) and HSQC spectra showed that **1** contained two ABX spin systems assignable to two 1,3,4-trisubstituted benzene rings at *δ*_H_ 6.64 (1H, *d*, *J* = 8.0 Hz, H-5), 6.68 (1H, brs, H-6), and 6.82 (1H, brs, H-2); and 6.69 (1H, brs, H-5′), 6.62 (1H, dd, *J* = 8.0, 1.4 Hz, H-6′), and 6.73 (1H, d, *J* = 1.4 Hz, H-2′).

**Table 1:** ^1^H- (600 MHz) and ^13^C-NMR (150 MHz) data of compound **1** (in CD_3_OD, *δ* in ppm, *J* in Hz).

No.	*δ*_H_	*δ*c	No.	*δ*_H_	*δ*c
1	-	134.4	8′	2.62 m	42.1
2	6.82 brs	110.1	9′	3.54 dd (7.6, 7.6), 3.82 dd (7.5, 7.5)	72.3
3	-	147.7	C-3-OMe	3.72 s	55.6
4	-	145.5	C-3′-OMe	3.72 s	55.6
5	6.64 d (8.0)	115.3	Glc-1′′	4.32 d (7.7)	101.4
6	6.68 brs	118.7	2′′	3.30 m	81.9
7	4.76 d (6.4)	81.7	3′′	3.13 m	76.2
8	2.31 m	50.1	4′′	3.13 m	69.7
9	3.64 m, 3.88 dd (9.5, 6.4)	66.7	5′′	3.41 m	76.3
1′	-	131.8	6′′	3.44 m, 3.66 m	60.8
2′	6.73 d (1.4)	112.9	Glc-1′′′	4.43 d (7.8)	104.4
3′	-	147.3	2′′′	2.99 m	74.7
4′	-	144.5	3′′′	3.13 m	76.7
5′	6.69 brs	115.0	4′′′	3.03 m	69.9
6′	6.62 dd (8.0, 1.4)	120.8	5′′′	3.03 m	77.0
7′	2.82 dd (13.5, 4.3), 2.36 dd (13.0, 11.8)	32.4	6′′′	3.99 dd (9.5, 6.4), 3.58 m	61.1

**Figure 2:**
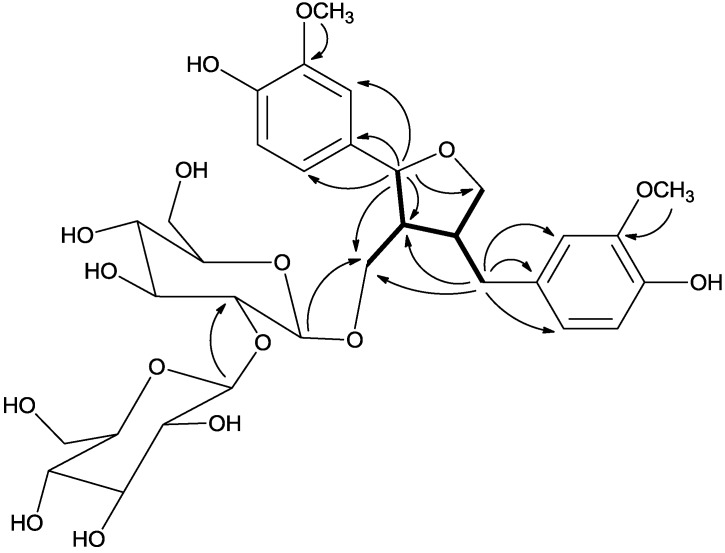
Key ^1^H-^1^H COSY (bold dash) and HMBC (arrow) correlations of **1**.

The ^1^H-NMR chemical shifts observed for these two benzene systems together with the presence of two oxygenated methyl proton signals at *δ*_H_ 3.72 (6H, s) suggested the presence of two guaiacyl (3-methoxy-4-hydroxyphenyl) groups in this compound [13]. The ^13^C-NMR assignments corroborated the ^1^H-NMR assignments for both aromatic rings. In addition, the ^1^H-NMR spectrum of **1** also established a C(7)-O-C(9′) tetrahydrofuran skeleton [14], similar to that of **4** [12]. The proton signals at *δ*_H_ 4.76 (1H, d, *J* = 6.4 Hz, H-7), 2.31 (1H, m, H-8), 3.88 (1H, dd, *J* = 9.5, 6.4 Hz, H-9a), 3.64 (1H, m, H-9b); and 2.82 (1H, dd, *J* = 13.5, 4.3 Hz, H-7′a), 2.36 (1H, dd, *J* = 13.0, 11.8 Hz, H-7′b), 2.62 (1H, m, H-8′), 3.82 (1H, dd, *J* = 7.5 Hz, 7.5 Hz, H-9′a) and 3.54 (1H, dd, *J* = 7.6 Hz, 7.6 Hz, H-9′b) were assigned to the substructures ^7^CH-^8^CH-^9^CH_2_O, ^7′^CH_2_-^8′^CH-^9′^CH_2_, and ^8^CH-^8′^CH from its ^1^H-^1^H COSY (Figure 2).

The ^1^H-NMR spectrum of **1** also gave signals for two glucose moieties (Table 1). In the HSQC spectrum, two anomeric protons at *δ*_H_ 4.32 (1H, d, *J* = 7.7 Hz, H-1′′) and 4.43 (1H, d, *J* = 7.8 Hz, H-1′′′), correlated respectively with signals at *δ*c 101.4 and 104.4. Both the anomeric protons appear as doublet signals with coupling constants around 8.0 Hz, consistent with axial orientations for these two monosaccharide moieties.

The ^13^C-NMR spectrum of **1** analyzed with the aid of DEPT spectrum showed the presence of two methoxy [*δ*c 55.6 (OMe-3, 3′)], three methylene [*δ*c 72.3 (C-9′), 66.7 (C-9) and 32.4 (C-7′)], three methine [*δ*c 81.7 (C-7), 50.1 (C-8) and 42.1 (C-8′)], twelve signals of two glucose moieties, and twelve aromatic carbons, of which six were quaternary (*δ*c 147.7, 147.3, 145.5, 144.5, 134.4, and 131.8), and the remaining six were unsubstituted aromatic carbons (*δ*c 120.8, 118.7, 115.3, 115.0, 112.9, and 110.1) (Table 1). All the information mentioned above substantiates the identity of **1** as a tetrahydrofuran lignan diglucoside [15,16].

The HMBC correlations between H-7 and C-1, C-2, and C-6 of the benzene ring indicated the linkage of C-7 and this aromatic ring. Similarly, C-7′ was connected to C-1′ of another aromatic ring as evidenced by the HMBC correlations between H-7′ and C-1′, C-2′ and C-6′ (Figure 2). The attachment of the inner glucose residue to C-9 was suggested by the HMBC correlation of the anomeric proton at *δ*_H_ 4.32 (d, *J* = 7.7 Hz) with the signal at *δ*_H_ 66.7 (C-9). The 1→2 interglycosidic linkage with the second glucose moiety was deduced from the HMBC correlation between H-1′′′ (*δ*_H_ 4.43, d, *J* = 7.8 Hz) and C-2′′ at *δ*c 81.9. The assignment of the 1→2 interglycosidic linkage of the two glucoses has also been confirmed by comparison with the ^13^C NMR spectral data of the reference [17].

**Figure 3:**
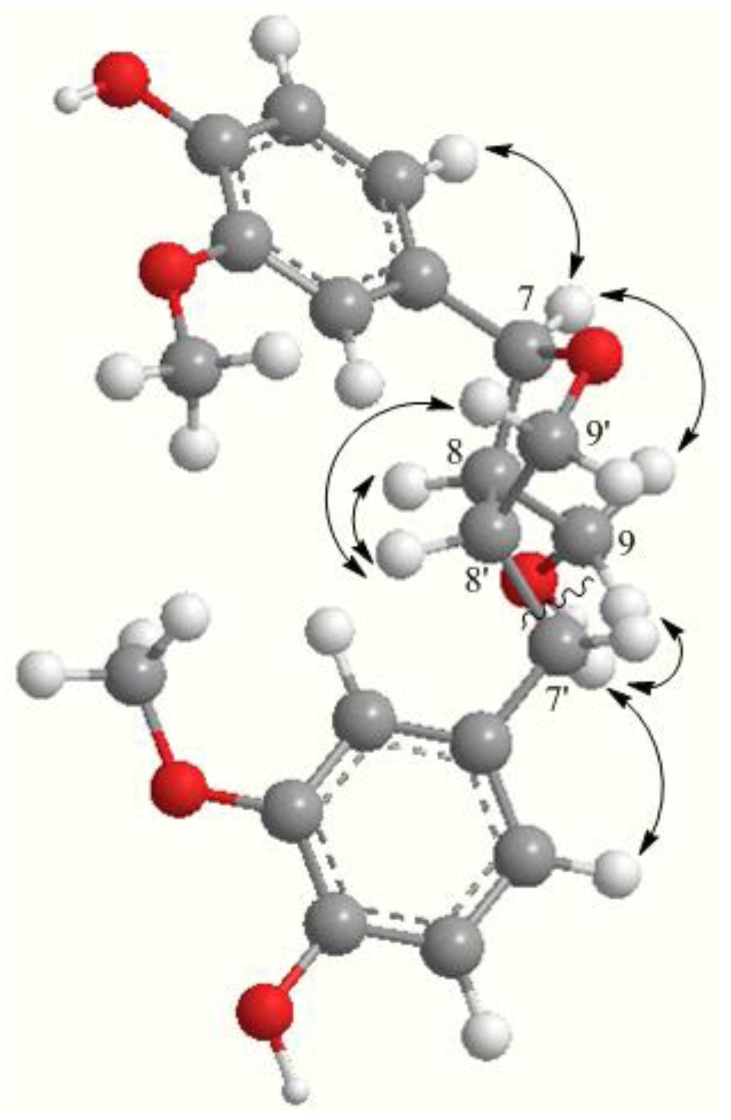
Key ROESY correlations (double-headed arrow) for the aglycone moiety of **1**.

The relative configuration of **1** was determined as described below. The ROESY spectrum displayed correlations between H-8 (*δ*_H_ 2.31) and H-8′ (*δ*_H_ 2.62) but was not observed between H-7 (*δ*_H_ 4.76) and H-8 (*δ*_H_ 2.31), leading to the assignments of *cis* and *trans* orientations for H-8/H-8′ and H-7/H-8, respectively (Figure 3).

In addition, a large coupling constant (*J* = 6.4 Hz) of H-7/H-8 [18] and the proton signal of H-7 at *ca.* 4.70 [19] further confirmed a *trans* configuration between the H-7 and H-8 protons. The absolute configuration of **1** was determined as 7*S*, 8*R*, 8′*R* by comparison of its optical rotation (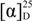 = +15.1°) with literature reports [19,20,21]. These data agreed well with the circular dichroism spectrum of **1**. The positive Cotton effect [Δε: + 4.35 (215 nm)] of **1** was in good agreement with that of (7*S,*8*R,*8′*R*)-5,5′-dimethoxylariciresinol 9′-*O*-β-d-glucopyranoside [Δε: +11.1 (211 nm)] [18] and the negative Cotton effect [Δε: −2.26 (291 nm)] of **1** was similar with that of (+)-lariciresinol 4′-*O*-β-d-glucopyranoside [Δε: −0.47 (280 nm)] [22] and (+)-lariciresinol 9′-stearate (289 nm) [23]. In summary, the structure of **1** was established as (7*S*,8*R*,8′*R*)-(+) lariciresinol 9-*O*-β-d-glucopyranosyl (1→2)-β-d-glucopyranoside, and named tianshanoside A. This new compound is a lariciresinol 9-*O*-diglucoside derivative, while its monoglucoside (lariciresinol 9-*O*-glucoside) had previously been isolated from the root of *Isatis indigotica* [24]. This is the first report of isolation of a lignan skeleton from the genus *Viola*.

The known compounds **2**–**4** were identified as syringin (**2**) [10], picraquassioside C (**3**) [11], and aketrilignoside B (**4**) [12] (Figure 1), respectively, by comparison of their NMR and MS data with those reported. All these compounds were reported for the first time from *V*. *tianshanica.* Compounds **1**, **3**, and **4** were tested *in vitro* for their cytotoxicities against two human tumor cell lines, gastric cancer Ags and liver cancer HepG2 cells, using the CCK-8 assay. None of the tested compounds showed any significant cytotoxic activities at concentrations up to 0.2 mM.

## 3. Experimental

### 3.1. General Procedures

NMR spectra were recorded on a Bruker DRX-400 or a DRX-600 spectrometer (Bruker, Fallanden, Switzerland) with TMS as an internal standard. ESI-MS spectra were obtained on an Agilent SL G1946D single quadrupole mass spectrometer (Agilent, Foster, CA, USA). HRESIMS was measured on a Bruker APEX III TESLA FTMS spectrometer (Bruker). Column chromatography was carried out on silica gel (200–300 mesh, Qingdao Marine Chemical Factory, Qingdao, China), Sephadex LH-20 (Pharmacia, Uppsala, Germany) and Chromatorex ODS (30-50 μm, Fuji Silysia Chemical Co. Ltd., Aichi, Japan). Semi-preparative HPLC separation was performed on an LC3000 system equipped with a P3000 pump, a UV3000 detector (Beijing Chuangxintongheng Science & Technology Co. Ltd., Beijing, China), and a Luna RP-18 column (250 mm × 10 mm, 10 μm, Phenomenex, Torrance, CA, USA). Optical rotation was determined on a Rudolph Autopol I polarimeter (Rudolph Research Analytical, Hackettstown, NJ, USA). Circular dichrosim spectroscopy was recorded on a Jasco 810 spectropolarimeter (Jasco Corporation, Tokyo, Japan).

### 3.2. Plant Material

The whole plant of *V. tianshanica* was collected from the Xinjiang Uygur Autonomous Region, China, in June 2012, and authenticated by Dr. Zhihong Cheng. A voucher specimen (NO. 201206TSJC) is deposited at the Herbarium of Materia Medica, School of Pharmacy, Fudan University, Shanghai, China.

### 3.3. Extraction and Isolation

The dried whole plant of *V. tianshanica* (20 kg) was extracted thrice with 70% aqueous ethanol (20 L) at room temperature for 4 h. The ethanolic extracts were combined and evaporated to dryness under reduced pressure, yielding a dark green residue (1,200 g). The residue was suspended in water (2 L) and partitioned successively with petroleum ether (60–90 °C), ethyl acetate, and *n*-butanol (2 L each) to yield the three corresponding extracts. The ethyl acetate extract (280 g) was fractionated on a silica gel column and eluted with increasing polarities of mixture of CH_2_Cl_2_ and MeOH to afford nine fractions A–I. Fraction E was subjected to repeated column chromatography on silica gel and eluted with a gradient solvent system of CH_2_Cl_2_-MeOH (50:1, 30:1, 20:1, 10:1, 5:1, 2:1, and 1:1). The fraction eluting with CH_2_Cl_2_-MeOH (10:1) was re-chromatographed on an ODS column eluted with H_2_O-MeOH (6:4), followed by semi-preparative HPLC using MeOH-H_2_O (15:85) as eluting solvents to give compound **2** (6.0 mg). Fraction F was separated by a silica gel column with a gradient solvent system of CH_2_Cl_2_-MeOH (50:1, 30:1, 20:1, 10:1, 5:1, 2:1, and 1:1). The 10:1 eluate was then purified by ODS column chromatography with MeOH-H_2_O (3:7) and semi-preparative HPLC (20% CH_3_CN) to give compounds **3** (25 mg) and **4** (50 mg). Fraction G was chromatographed on a Sephadex LH-20 column eluting with MeOH, followed by purification on a semi-preparative HPLC with MeOH-H_2_O (35:65) at a flow rate of 3 mL/min to give compound **1** (5.0 mg).

### 3.4. Spectral Data

*Tianshanoside A* (**1**). White amorphous powder, 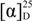 = +15.1° (*c* 0.20, MeOH); IR (KBr) ν_max_ 3,387 (HO), 2,924, 1,637, 1,513 (benzene rings), 1,452, 1,249, 1,121, 1,060, 764, 617 cm^−1^; UV (MeOH) λ_max_ 230, 280 nm; Circular dichrosim (MeOH): 215 (Δε + 4.35), 291 (Δε −2.26) nm; ^1^H- and ^13^C-NMR data, see Table 1; ESI-MS *m/z* 707 [M + Na]^+^; HRESIMS *m/z* 707.2531 [M + Na]^+^ (calcd for C_32_H_44_O_16_Na, 707.2521).

*Syringin* (**2**). Amorphous powder. ^1^H-NMR (CD_3_OD): δ_H_ 6.77 (*s*, H-2, 6), 6.57 (*d*, 15.8 Hz, H-7), 6.35 (*dt*, 15.8, 5.6 Hz, H-8), 4.24 (*d*, 5.6 Hz, H-9, 10), 3.88 (*s*, 3, 5-OMe). ^13^C-NMR (CD_3_OD): δ_C_ 154.3 (C-3, 5), 135.2 (C-1), 131.2 (C-7), 130.0 (C-8), 105.4 (C-2, 6), 105.3 (C-1′), 78.3 (C-5′), 77.8 (C-3′), 75.7 (C-2′), 71.3 (C-4′), 63.5 (C-9), 62.5(C-6′). ESI-MS: *m*/*z* 395 [M + Na]^+^, 411 [M + K]^+^.

*Picraquassioside C* (**3**). Amorphous powder. ^1^H-NMR (CD_3_OD): δ_H_ 6.81 (*d*, 1.5 Hz, H-2′, 6′), 6.73 (*d*, 1.5 Hz, H-3′′, 5′′), 6.55 (*br d*, 15.9 Hz, H-1′′′), 6.35 (*dt*, 15.9, 5.5 Hz, H-2′′′), 5.03 (*d*, 5.4 Hz, H-1), 4.80 (*d*, 7.3 Hz, H-1-Glc), 4.25 (*m*, H-2, 3′′′a, 3′′′b), 3.86 (*s*, 3′, 5′-OMe), 3.85 (*s*, 3′′, 5′′-OMe), 3.2–3.6 (*m*, H-2-6-Glc, H-3). ^13^C-NMR (CD_3_OD): δ_C_ 154.2 (C-2′′, 6′′), 153.7 (C-3′, 5′), 139.2 (C-1′), 136.8 (C-1′′), 135.4 (C-4′), 134.7 (C-4′′), 131.2 (C-2′′′), 129.9 (C-1′′′), 105.7 (C-2′, 6′), 105.6 (C-1-Glc), 104.7 (C-3′′, 5′′), 87.9 (C-2), 78.3 (C-5-Glc), 78.1 (C-3-Glc), 75.7 (C-2-Glc), 74.0 (C-1), 71.3 (C-4-Glc), 63.5 (C-3′′′), 62.5 (C-6-Glc), 61.9 (C-3), 56.9 (C-3′, 5′-OMe), 56.6 (C-3′′, 5′′-OMe). ESI-MS: *m*/*z* 621 [M + Na]^+^, 637 [M + K]^+^.

*Aketrilignoside B* (**4**). Colorless gum. ^1^H-NMR (CD_3_OD): δ_H_ 7.36 (*s*, H-2′, 6′), 6.78 (*s*, H-2, 6), 4.84 (*d*, 8.7 Hz, H-7), 4.38 (*m*, H-8′), 4.30 (*d*, 7.8 Hz, H-1-Glc), 4.26 (*m*, H-9′a), 4.21 (*m*, H-9′b), 4.09 (*dd*, 10.3, 4.1 Hz, H-9a), 3.94 (*s*, 3′, 5′-OMe), 3.88 (*s*, 3, 5-OMe), 3.62 (*m*, H-9a), 3.20-3.85 (*m*, H-2-6-Glc), 2.75 (*m*, H-8). ^13^C-NMR (CD_3_OD): δ_C_ 200.5 (C-7′), 149.3 (C-3, 5), 149.2 (C-3′, 5′), 142.9 (C-4′), 136.3 (C-4), 132.6 (C-1), 128.7 (C-1′), 107.8 (C-2′, 6′), 105.3 (C-2, 6), 105.1 (C-1-Glc), 85.3 (C-7), 78.2 (C-3-Glc), 78.1 (C-5-Glc), 75.2 (C-2-Glc), 71.7 (C-9′), 71.6 (C-4-Glc), 69.1 (C-9), 62.8 (C-6-Glc), 57.1 (C-3′, 5′-OMe), 56.8 (C-3, 5-OMe), 53.3 (C-8), 50.1 (C-8′). ESI-MS: *m*/*z* 597 [M + H]^+^, 619 [M + Na]^+^.

### 3.5. Cytotoxicity Evaluation

Two human cancer cell lines, the gastric cancer Ags cells and HepG2 liver cancer cells were obtained from the Cell Bank of the Chinese Academy of Sciences (Shanghai, China). The Ags cell line was cultured in a Ham’s/F12 medium (Hyclone, Logan, UT, USA), supplemented with 10% fetal bovine serum (Gibco). The HepG2 cell line was cultured in DMEM medium (Hyclone), supplemented with 10% fetal bovine serum (Gibco). Cells were maintained at 37 °C in a 95% air-5% CO_2_ atmosphere. The cytotoxicity of the compounds was determined *in vitro* using the Cell Counting Kit-8 (CCK-8) assay kit (Dojindo Laboratories, Tokyo, Japan), according to the manufacturer′s instructions. The sample compounds were dissolved in DMSO, and then further diluted. Cells were plated at a density of 1.0 × 10^5^ per well in a 96-well microplate and incubated at 37 °C for 24 h. The cells were treated with various concentrations of test compounds (the final concentrations of the compounds were 0, 25, 75, 100, 150, 200 μM/mL) in quadruplicate for 72 h. Then, 20 μL of the solution of CCK-8 was added into each well, and the plates were further incubated for an additional 1 h. The absorbance was measured at 450 nm with a Varioskan Flash microplate reader (Thermo Scientific, Waltham, MA, USA).

## 4. Conclusions

Three lignan glycosides including a new one, tianshanoside A, and a phenylpropanoid glycoside were isolated for the first time from the dried whole plant of *V. tianshanica*. This is the first report of lignans from *Viola* species. Three lignan glycosides exhibited no significant inhibitory activities against two human tumor cell lines, gastric cancer Ags or liver cancer HepG2.
